# Hormonal, follicular and endometrial dynamics in letrozole-treated versus natural cycles in patients undergoing controlled ovarian stimulation

**DOI:** 10.1186/1477-7827-9-83

**Published:** 2011-06-21

**Authors:** Mohamed A Bedaiwy, Mahmoud A Abdelaleem, Mostafa Hussein, Noha Mousa, Lisa N Brunengraber, Robert F Casper

**Affiliations:** 1 Department of Obstetrics and Gynecology, University Hospitals Case Medical Center, Case Western Reserve University School of Medicine, Cleveland, Ohio, USA; 2 Assiut University, Assiut, Egypt; 3 Reproductive Sciences Division, Department of Obstetrics and Gynecology, University of Toronto, Toronto, Canada

## Abstract

The objective of this study was to compare letrozole-stimulated cycles to natural cycles in 208 patients undergoing intrauterine insemination (IUI) between July of 2004 and January of 2007. Group I (n = 47) received cycle monitoring only (natural group), Group II (n = 125) received letrozole 2.5 mg/day on cycle days three to seven, and Group III (n = 36) received letrozole 5 mg/day on cycle days three to seven. There were no differences between the groups in endometrial thickness or P_4 _on the day of hCG. Estradiol levels had higher variation in the second half of the follicular phase in both letrozole-treated groups compared to the control group. Estradiol per preovulatory follicle was similar in both letrozole cycles to that observed in the natural cycles. LH was lower on the day of hCG administration in the letrozole 2.5 mg/day group vs. the natural group. In summary, letrozole results in some minor changes in follicular, hormonal and endometrial dynamics compared to natural cycles. Increased folliculogenesis and pregnancy rates were observed in the letrozole-treated groups compared to the natural group. These findings need to be confirmed in larger, prospective studies.

## Background

For over 40 years, the first-line therapy for ovulation induction (OI) has been clomiphene citrate (CC) [1]. Its inherent properties such as low price, oral route of administration and high ovulation success rate (60-90%) make it an attractive therapy. However, the pregnancy rate is [2] disappointing. Sub-optimal pregnancy rates with CC have been attributed to peripheral anti-estrogenic effects, mainly on the endometrium and the cervical mucus[3] Gonadotropins are more effective in ovulation induction and are associated with higher pregnancy rates than CC, but are expensive and carry higher risk for ovarian hyperstimulation syndrome and multiple gestations [4].

Newer options for ovulation induction are the third-generation aromatase inhibitors (AIs), the most commonly used being letrozole. Initially introduced to treat postmenopausal breast cancer, AIs are now also being used for ovulation induction or enhancement. A recent meta-analysis addressing the use of letrozole in assisted conception concluded that letrozole is as effective as other methods of ovulation induction [5]. When letrozole is used in combination with gonadotropins, it leads to lower gonadotropin requirements and pregnancy rates similar to gonadotropin treatment alone [6]. In a study comparing combined therapy of letrozole (2.5 mg/day or 5.0 mg/day) and recombinant FSH with recombinant FSH alone in an intrauterine insemination (IUI) program, 5 mg/day of letrozole was more cost-effective than the 2.5 mg/day in co-treatment with no adverse effect on pregnancy rate or outcome [7].

Aromatase inhibitors for ovulation induction are orally administered, and are relatively inexpensive with minor side effects such as very infrequent headaches and leg cramps[8]. Aromatase inhibitors increase endogenous FSH production in response to decreased estrogen biosynthesis in the ovary and extraovarian tissues, including the brain [9]. Because they do not deplete estrogen receptors like CC, normal central feedback mechanisms remain intact[10]. As the dominant follicle grows and estrogen levels rise, normal negative feedback occurs centrally resulting in suppression of FSH and atresia of the smaller growing follicles. Therefore, a single dominant follicle, and mono-ovulation, is the rule in most cases[11] with the clear advantage of reducing multiple-gestation pregnancies.

Compared to CC, letrozole has been associated with lower preovulatory estradiol (E_2_) levels [12], as well as thicker endometrium and a trend towards higher pregnancy rates[13]. Standard ovarian stimulation protocols often produce high preovulatory E_2 _levels that could adversely affect the development of the endometrium, the follicles, and the embryo. Therefore, the lower E_2 _when using AIs may lead to an improvement of implantation [14,15].

There have been several studies comparing letrozole to CC. However, there is a paucity of research comparing letrozole with natural cycles. Larger studies comparing CC, letrozole, and natural cycles in a single study are necessary to further characterize the effect of letrozole on hormonal dynamics and pregnancy rates. Our hypothesis is that letrozole-treated cycles mimic natural cycles in hormonal and endometrial parameters. The aim of this study was to compare cycle dynamics in letrozole-treated versus natural cycles in infertile patients undergoing intrauterine insemination (IUI).

## Methods

### Patient recruitment and counseling

We conducted a retrospective cohort study of 208 consecutive infertile patients who were recruited to participate. Briefly, patients underwent IUI between July of 2004 and January of 2007 at the Toronto Center For Assisted Reproductive Technology, Toronto, Canada. Patients were divided into three groups. The first group (n = 47) received cycle monitoring only (natural cycle; group I). The 2 remaining groups received letrozole on cycle days three to seven at either 2.5 mg/day (n = 125) (group II) or 5 mg/day (n = 36) (group III). An informed consent was obtained from all participants clearly denoting the off-label use of the medication prior to treatment. Subsequently IRB approval was obtained to use their data for the purposes of this study.

Causative factors of infertility were investigated and defined as follows. Tubal patency was confirmed by sonohysterography with contrast, hysterosalpingography, and or pelvic laparoscopy. Mild male factor infertility was diagnosed according to the World Health Organization (WHO) criteria for normal semen[16]. Endometriosis was diagnosed by pelvic laparoscopy. Unexplained infertility was based on the exclusion of known factors of infertility.

### Cycle monitoring and insemination

All patients were followed with serial measurements of serum estradiol (E_2_), progesterone (P_4_), and luteinizing hormone (LH) using a radioimmunoassay (RIA) kit. Transvaginal ultrasonography (TVS) was performed for follicular diameter tracking and measurement of endometrial thickness. We measured follicular diameter in 2 perpendicular planes and calculated the mean, while endometrial thickness was measured in the sagittal plane at the widest part of endometrial cavity. Serum samples were obtained and TVS performed on cycle days three, seven, once between days 9 and 11, the day of human chorionic gonadotropin (hCG) administration, and when a follicle achieved a diameter of ≥16 mm. Serum follicle stimulating hormone (FSH) was measured on day 3 only. The results for E_2 _and P_4 _levels were reported as picomoles per liter and nanomoles per liter, respectively, and LH and FSH levels were reported in international units per liter.

An LH surge was defined as an increase in LH level greater than 100% over the mean of the preceding 2 measures. IUI was performed 36-40 hours after hCG administration if no endogenous LH surge occurred. If an endogenous LH surge was detected on the day of hCG administration, two IUIs were performed at 24 and 48 h. Pregnancy was diagnosed by quantitative β-hCG assay two weeks after the insemination. Clinical pregnancy was confirmed by observing fetal cardiac pulsation four weeks after positive pregnancy test by TVS.

### Outcome parameters

Hormonal outcome measures were E_2_, P_4_, LH and FSH. Non-hormonal outcomes were number and size of growing follicles, endometrial thickness, and pregnancy. All three treatment groups were compared to each other for hormonal, endometrial and follicular dynamics, while the two groups receiving letrozole were combined for comparison against the natural cycle group for pregnancy rates.

### Statistical analysis

Data management was done using a preprepared Excel data spreadsheet. Outcome measures are expressed as mean ± standard deviation from the mean (SD). Statistical significance for continuous variables was calculated using ANOVA test. Categorical variables were compared using the *χ*^2 ^and Fisher's Exact Test. P < 0.05 was considered statistically significant. Statistical analysis was performed with SPSS (Release 14.01, SPSS Inc., Chicago, IL).

## Results

### Patients

The study included 208 patients who underwent a total of 300 consecutive IUI cycles. There were 71 cycles in the natural cycle group, 179 cycles in the letrozole 2.5 mg/day group, and 50 cycles in the letrozole 5 mg/day group (table 1). Male factor and unexplained infertility were the most common indications for IUI. Other identified causes of infertility included endometriosis, and polycystic ovarian syndrome. The most frequent cause of cycle cancellation differed among the patient groups. The most common cause of cancellation in the natural cycle group was anovulation, while in the letrozole 2.5 mg/day group, the main cause was presence of ovarian cysts on cycle day 3 before the start of stimulation. The most common cause of cancellation in the letrozole 5 mg/day group was an elevated serum FSH on cycle day 3 before the start of stimulation.

**Table 1:** Study demographics

	*Group I (Natural cycle)*	*Group II (letrozole 2.5 mg)*	*Group III (letrozole 5 mg)*	*P value*
**Total No of patients**	47	125	36	
**Age (Mean ± SD)**	34.8 ± 5.03	33.5 ± 4.02	33.88 ± 3.45	NS
**Day 3 FSH (IU/L)**	6.01 ± 1.71	6.47 ± 2.16	7.49 ± 3.76	I ***Vs ***II: 0.233 I ***Vs ***III: 0.033
**Total No of cycles started**	71	179	50	
**Completed cycle: No, [%]**	63 [88.7]	142 [79.3]	43 [86]	
**Total number of cycles cancelled: No, [%]**	8 [11.3]	37 [20.7]	7 [14]	0,06
**No of monitoring visits (Mean ±SD)**	2.08 ± 1.02	2.3 ± 1.12	2.68 ± 1.11	I ***Vs ***II: 0.232 I ***Vs ***III: 0.003
**Causes of cycle cancelling**				
Poor responder: No, [%]	5 [62.5]	9 [24.3]	0 [0]	NS
Ovarian cyst: No, [%]	0 [0]	16 [43.2]	2 [28.6]	NS
Patient request: No, [%]	1 [12.5]	8 [21.6]	0 [0]	NS
Elevated FSH: No, [%]	0 [0]	2 [5.4]	3 [42.9]	NS
Others: No, [%]	2 [25]	2 [5.4]	2 [28.6]	NS
**Indication for IUI**				
Male factor infertility: No, [%]	38/71 [53.5]	55/179 [30.7]	19/50 [38]	NS
Unexplained infertility: No, [%]	33/71 [46.5]	106/179 [59.3]	30/50 [60]	NS
Endometriosis: No, [%]	0 [0]	9/179 [5]	0 [0]	NS
Others No, [%]	1/71 [1.4]	9/179 [5]	1 [2]	NS

### Hormonal dynamics

Values for E_2_, P_4 _and LH are shown in table 2. The letrozole 5 mg/day group had significantly lower E_2 _on day seven, but significantly higher E_2 _on the day of hCG administration when compared to the natural group (P = 0.025 and 0.041, respectively). However, the E_2 _per preovulatory follicle was similar in all three groups on the day of hCG. On days 9 to 11 and the day of hCG administration, E_2 _had a larger variability, as evidenced by larger standard deviations, in the letrozole groups compared to the natural group (Table 2). LH was significantly lower on the day of hCG administration in the letrozole 2.5 mg/day group compared to the natural group (p = 0.000). P_4 _showed no differences between any treatment groups.

**Table 2:** Hormonal dynamics: follicular phase levels of serum estradiol, progesterone, and LH

	Estradiol (pmol/mL)			Progesterone (pmol/mL)			LH (IU/L)		
	Group I (Natural cycle)	Group II letrozole 2.5 mg	Group III (letrozole 5 mg)	Group I (Natural cycle)	Group II (letrozole 2.5 mg	Group III (letrozole 5 mg)	Group I (Natural cycle)	Group II (letrozole 2.5 mg	Group III (letrozole 5 mg)
D3	139.8 ± 66.36	154.22 ± 131.31	126.71 ± 46.75	2.35 ± 0.84	2.57 ±.90	2.55 ±.89	5.18 ± 2.25	5.22 ± 3.41	5.28 ± 2.71
D7	257.75 ± 187.8	196.60 ± 127.07	147.63 ± 69.74	2.08 ± 0.40	2.37 ± 0.87	2.80 ± 0.82	5.1 ± 1.34	6.71 ± 4.23	8.28 ± 4.07
D 9-11	380.48 ± 238.78	810.05 ± 178.26	562.53 ± 137.51	2.35 ± 0.85	2.2 ± 0.79	2.67 ± 0.47	5.89 ± 2.96	5.76 ± 3.30	6.65 ± 2.95
D hCG	911.48 ± 941.96	1275.41 ± 927.66	1066.16 ± 1876.37	2.46 ± 1.32	2.40 ± 0.89	2.75 ± 0.75	17.39 ± 16.09	8.92 ± 9.86	10.50 ± 13.81
E2/follicle ≥ 16 mm	593.12 ± 577.4	584.41 ± 1147.84	389.8 ± 849.79						

P value: 0.025: day7 for serum Estradiol between group I and III.

P value: 0.041 day of hCG For serum Estradiol between group I and III.

P value: 0.000 LH at day of HCG group I vs II

### Follicular dynamics

There were no statistically significant differences in follicular diameter between the three groups until cycle day seven (table 3).Compared to the natural group, both the letrozole 2.5 mg/day and 5 mg/day groups had significantly more follicles ≥ 10 mm on day 7 (p = 0.0001 and 0.0001, respectively), more follicles ≥ 12 mm on days 9-11 (p = 0.0001 and 0.005), and more follicles ≥ 15 mm on the day of hCG administration (p = 0.0001 and 0.0001).

**Table 3:** Follicular dynamics in study groups

	Group I	Group II	Group III	P value
**Day 3 diameter of follicles in cm**	0.56 ± 1.02	0.79 ± 1.12	0.53 ± 0.86	I vs III: 0.9
				I vsII: 0.26
**Number of D 7-8 follicles ≥ 10 mm**	0.80 ± 0.63	2.52 ± 1.37	2.23 ± 1.48	I vs III: *0.000*
				I vsII: *0.000*
**Number of D 9-11follicles more than 12 mm**	1.13 ± 0.73	2.09 ± 1.00	2.29 ± 1.35	I vs III: *0.005*
				I vsII: *0.000*
**Number of mature follicles (≥15 mm) at the Day of hCG**	1.20 ± 0.48	1.84 ± 0.92	2.12 ± 0.99	I vs III: *0.000*
				I vsII: *0.000*

### Endometrial dynamics

When comparing endometrial thickness, neither letrozole group differed significantly from the natural group on any day (table 4).

**Table 4:** Endometrial thickness (cm) in study groups

	Group I	Group II	Group III
**Cycle day 3**	0.14 ± 0.26	0.10 ± 0.23	0.15 ± 0.24
**Cycle day 7**	0.55 ± 0.26	0.56 ± 0.19	0.50 ± 0.19
**Cycle day 9-11**	0.65 ± 0.18	0.77 ± 0.96	0.62 ± 0.16
**Day of hCG injection**	0.86 ± 0.16	0.82 ± 0.14	0.86 ± 0.23

***P ***value non-significant when comparing group I to either group II or III.

### Pregnancy rate

Three out of 63 natural cycles (4.8%) and 22 out of 185 letrozole cycles (12%) resulted in pregnancy (table 5). This difference was statistically significant (p = = 0.02). Although only a small number of cycles were repeated as part of this study, the cumulative pregnancy rate was 13.7% in the letrozole groups, significantly higher than the natural group rate of 6.4% (p = 0.01). There were 3 twin pregnancies in the 2 letrozole groups and none in the natural cycle group.

**Table 5:** Pregnancy rate in study groups

	*Group I Number (%)*	*Combined groups II, III Number (%)*	P *value*
**Pregnancy rate/cycle started**	3/71 (4.2)	22/229 (9.61)	**0.02**
**Pregnancy rate/cycle completed**	3/63 (4.8)	22/185 (11.89)	**0.02**
**Cumulative pregnancy rate**	3/47 (6.4)	22/161 (13.7)	**0.01**
**Twin pregnancy**	0/3 (0)	3/22 (13.6)	**0.01**
**Miscarriage (No)**	0/3 (0)	3/22 (13.6)	**0.01**

## Discussion

Endometrial sparing, increased folliculogenesis, and an increase in pregnancy rate were observed in our letrozole-treated patients as compared to naturally-cycling patients. The endometrial-sparing effect of letrozole is well-demonstrated in this study by the lack of significant difference in endometrial thickness compared to the natural cycle group, and also has been confirmed by previous studies [17,18]. In addition, our ovulation monitoring findings agree with a previous study showing multifollicular development and better pregnancy outcomes with the use of letrozole 5 mg/day [14,17]. Our finding that serial serum progesterone levels were comparable between the three groups agrees with a previous study [18], and suggests that letrozole does not have a premature luteinizing effect on the developing follicle. Table 6 summarizes the results of some trials using letrozole as an ovulation inducing agent in different clinical scenaria with different results and conclusions.

**Table 6:** Summary of randomized trials assessing the efficacy of letrozole

Study (reference number)	Intervention	Cohort of patients	Conclusion
[20]	Letrozole (2.5 mg) Vs CC	Infertile women undergoing Superovulation and IUI.	Similar endometrial thickness and pregnancy rates.
[21]	letrozole Vs CC as adjuvants to rFSH 41 patients	Superovulation before IUI in unexplained infertility	Better endometrial thickness with letrozole. Similar pregnancy rate
[22]	Letrozole Vs CC 74 patients	Polycystic ovary syndrome	Similar endometrial thickness and pregnancy rate
[23]	Letrozole (2.5 mg) Vs CC	Polycystic ovary syndrome	Better endometrial thickness and pregnancy rate with letrozole.
[24]	Letrozole Vs CC	Polycystic ovary syndrome	No advantage to the use of letrozole over CC as a first-line treatment for induction of ovulation in women with PCOS
[25,26]	Letrozole Vs CC	Superovulation before IUI in unexplained infertility	No superiority between letrozole and CC for inducing ovulation in women with unexplained infertility before IUI.
[27]	Letrozole Vs CC-gonadotropin	Superovulation before IUI in unexplained infertility	Letrozole is a good alternative to CC-gonadotropin.
[28]	Letrozole (2.5 mg) Vs CC 22 patients	superovulation in women with normal ovulation	CC is superior to 2.5 mg letrozole for superovulation induction in women with normal ovulation.
[29]	Letrozole (7.5 mg) Vs CC 46 patients	Polycystic ovary syndrome	Letrozole has better ovulation and PR in comparison to CC in patients with PCOS

Although basal LH levels were comparable among the 3 groups, there was a trend towards higher LH (although non-significant) on day 7 in the letrozole groups. This rise might be due to the release of the anterior pituitary from the negative feedback of E_2_. Three important observations deserve mentioning. First, the increase in LH is still well-below the levels for definition of premature LH surge. Second, after discontinuation of letrozole (days 9-11), serum LH returned to levels similar to the non-stimulated group reflecting the short half life of the letrozole. Thirdly, a natural LH surge was observed more frequently in non-stimulated cycles than in the letrozole stimulated cycles. This finding is likely artifactual since we suggest hCG administration to all women undergoing IUI so that timing of insemination can be optimized. The women undergoing natural cycle monitoring usually requested everything to be natural including no hCG trigger so the difference between the groups is unlikely to be related to the letrozole. Spontaneous LH surges do occur with letrozole for ovulation induction.

This study has some limitations, the most important of which include that it is retrospective, non-powered, non-randomized and not blinded. These issues are inherent in retrospective studies. However, we believe the results are of interest since there are few studies comparing natural cycles to letrozole-stimulated cycles. The main distinguishing features of letrozole as an ovulation inducing agent is its endometrial sparing effect and the early cycle multi-follicular development that may have translated into a better pregnancy rate in the present study. In addition, a recent study demonstrated that letrozole improves blood flow compared to CC and this observation may also be associated with improved pregnancy rates [19]. A well-designed and powered randomized clinical trial will be needed to confirm this result.

The authors declare that they have no competing interests.

## Competing interests

The authors declare that they have no competing interests.

## Authors' contributions

MAB collected and analyzed the data and drafted the manuscript. MAA Collected the data and drafting the manuscript. MH participated in the data collection. NM participated in the design of the study and performed the statistical analysis. LNB: participated in the data collection and helped to draft the manuscript. RC Conceived the idea of the study, and participated in its design and coordination and helped to draft the manuscript. All authors read and approved the final manuscript.
